# Response to experimental cold-induced pain discloses a resistant category among endurance athletes, with a distinct profile of pain-related behavior and GABAergic EEG markers: a case–control preliminary study

**DOI:** 10.3389/fnins.2023.1287233

**Published:** 2024-01-15

**Authors:** Franziska Peier, Michael Mouthon, Michael De Pretto, Joelle Nsimire Chabwine

**Affiliations:** ^1^Laboratory for Neurorehabilitation Science, Medicine Section, Faculty of Science and Medicine, University of Fribourg, Fribourg, Switzerland; ^2^Neurology Division, Department of Internal Medicine, Fribourg-Cantonal Hospital, Fribourg, Switzerland

**Keywords:** physical exercise, endurance training, pain, cold pressure test, electroencephalogram, GABA, pain resistance, exercise-induced hypoalgesia

## Abstract

Pain is a major public health problem worldwide, with a high rate of treatment failure. Among promising non-pharmacological therapies, physical exercise is an attractive, cheap, accessible and innocuous method; beyond other health benefits. However, its highly variable therapeutic effect and incompletely understood underlying mechanisms (plausibly involving the GABAergic neurotransmission) require further research. This case–control study aimed to investigate the impact of long-lasting intensive endurance sport practice (≥7 h/week for the last 6 months at the time of the experiment) on the response to experimental cold-induced pain (as a suitable chronic pain model), assuming that highly trained individual would better resist to pain, develop advantageous pain-copying strategies and enhance their GABAergic signaling. For this purpose, clinical pain-related data, response to a cold-pressor test and high-density EEG high (Hβ) and low beta (Lβ) oscillations were documented. Among 27 athletes and 27 age-adjusted non-trained controls (right-handed males), a category of highly pain-resistant participants (mostly athletes, 48.1%) was identified, displaying lower fear of pain, compared to non-resistant non-athletes. Furthermore, they tolerated longer cold-water immersion and perceived lower maximal sensory pain. However, while having similar Hβ and Lβ powers at baseline, they exhibited a reduction between cold and pain perceptions and between pain threshold and tolerance (respectively −60% and − 6.6%; −179.5% and − 5.9%; normalized differences), in contrast to the increase noticed in non-resistant non-athletes (+21% and + 14%; +23.3% and + 13.6% respectively). Our results suggest a beneficial effect of long-lasting physical exercise on resistance to pain and pain-related behaviors, and a modification in brain GABAergic signaling. In light of the current knowledge, we propose that the GABAergic neurotransmission could display multifaceted changes to be differently interpreted, depending on the training profile and on the homeostatic setting (e.g., in pain-free versus chronic pain conditions). Despite limitations related to the sample size and to absence of direct observations under acute physical exercise, this precursory study brings into light the unique profile of resistant individuals (probably favored by training) allowing highly informative observation on physical exercise-induced analgesia and paving the way for future clinical translation. Further characterizing pain-resistant individuals would open avenues for a targeted and physiologically informed pain management.

## 1 Introduction

Pain, especially chronic pain (CP), constitutes a major public health issue worldwide, affecting millions of individuals (Treede et al., 2015). Despite remarkable progress made in the development of analgesic drugs, about one third of pain-affected people do not experience satisfactory pain relief (Todd, 2017; Finnerup et al., 2018). This failure to obtain efficient pharmacological analgesia has brought interest and focus to numerous non-pharmacological methods such as mindfulness, hypnosis, physical therapy, etc. Among them, physical exercise (PE) is attractive, owing to its low cost, high accessibility and multiple beneficial effects on health, well beyond analgesia, e.g., in cardiovascular (Adams and Linke, 2019) and mental diseases (Schuch and Vancampfort, 2021), and in dementia (Jia et al., 2019 etc).

However, as for a number of other non-pharmacological methods, mechanisms counteracting pain in PE are not fully elucidated (Lesnak and Sluka, 2020). On the other hand, CP syndromes are usually not studied and/or managed according to their underlying pathological mechanisms (Vargas-Schaffer, 2010; Raffaeli et al., 2021). In fact, this mechanism-based approach of both CP syndromes and of analgesic therapies could be used as a strategy to improve pain management (Gallagher, 2006; Vardeh et al., 2016; Teixeira et al., 2021). Furthermore, by selecting the most responsive patients to therapies targeting a given pain mechanism, this “mechanism-targeted analgesia” would plausibly also reduce the variability of the therapeutic response.

Nevertheless, an appropriate study of mechanisms subtending the analgesic effect of PE in CP patients is subject to several challenges mainly related to patients’ reluctance to move and in particular to perform physical activities, as a consequence of aberrant behavioral changes (e.g., fear-avoidance, pain catastrophizing) developed toward pain and, in several of them, toward movement (Vlaeyen and Linton, 2000; Crombez et al., 2012; Zale and Ditre, 2015). On the other side, acute exercise is known to consistently reduce experimental pain in healthy individuals, although this effect remains brief and highly variable (Naugle et al., 2012; Koltyn et al., 2014; Vaegter and Jones, 2020). Regular exercise practiced over a long period, in turn, modifies the ability of pain-free individuals to cope with experimentally induced pain, which results, for example, in higher pain tolerance (Ellingson et al., 2012; Sluka et al., 2018; Vaegter and Jones, 2020). Furthermore, the so-called exercise-induced hypoalgesia (EIH) is more consistent in healthy individuals than in CP patients (Ellingson et al., 2016; Rice et al., 2019; Vaegter and Jones, 2020) under experimental pain conditions. For all these reasons, studying how PE modifies experimental pain response in pain-free populations could be an interesting alternative method to better understand physiological mechanisms subtending the analgesic effect of PE.

The current literature suggests that the analgesic effect of PE goes beyond purely psychological aspects (Lima et al., 2017; Sluka et al., 2018). The Gamma-Aminobutyric Acid (GABA)ergic signaling is impaired in neuropathic pain animal models (Senba and Kami, 2020). Upon PE, glutamate acid decarboxylase (GAD), the GABA-synthetizing enzyme, is upregulated, which results not only in GABAergic restoration, but also in reduced experimental nociception (Kami et al., 2016). Moreover, it seems that regular PE would counteract CP also by promoting balance between excitatory and inhibitory neurotransmission through improved GABAergic neurotransmission (Fitzgerald and Carter, 2011), possibly through enhancement of descending inhibitory pathways (Zheng et al., 2021).

In addition to the above-mentioned direct effect, regular PE is suggested to counteract central sensitization (Nijs et al., 2014; Tan et al., 2022). The latter represents originally a physiological adaptive process protective against (acute) pain (Latremoliere and Woolf, 2009), consisting in lowered activation threshold and facilitated response to painful stimuli of nociceptive pathway components in the central nervous system. However, as pain persists, central sensitization may become maladaptive and participate to the transition from acute pain to CP (Latremoliere and Woolf, 2009; Lluch et al., 2014; Nijs et al., 2016; Ji et al., 2018; Guler et al., 2020). Clinically, pathological central sensitization manifests as allodynia and hyperalgesia, while pain-related behaviors lose their protective benefit and become aberrant, now contributing to perpetuate pain (Latremoliere and Woolf, 2009; Jensen and Finnerup, 2014). Mechanistically, there is an increased excitability and facilitation (resulting from the imbalance between excitatory and inhibitory neurotransmission) that reasonably allows hypothesizing on, among other mechanisms, a deficit in GABAergic neurotransmission (Curatolo et al., 2006; Latremoliere and Woolf, 2009; Li et al., 2019a; Comitato and Bardoni, 2021; Wang et al., 2021).

Overall, the potential (direct or indirect) involvement of the GABAergic neurotransmission in exercise-induced analgesia is interesting and even physiologically meaningful in many regards, notably given the key role played by GABA in nociception, pain regulation and modulation (Enna and McCarson, 2006; Barr et al., 2013; Du et al., 2017; Li et al., 2019b; Wang et al., 2021; Benke, 2022), but also taking into account the GABAergic reduction documented in CP patients (Barr et al., 2013; Teixeira et al., 2021; Makowka et al., 2023). Thus, the GABAergic system could be a suitable target in the mechanistic approach of EIH. However, little is known about the GABAergic correlates of exercise-induced modifications of experimental pain response in humans. Also, behavioral modifications leading to the observed improvement in pain-copying strategies (i.e., higher tolerance; see above) are poorly documented.

This study aimed at investigating experimental pain response and its GABAergic correlates in pain-free individuals with different PE regimes, as an interesting strategy to study mechanisms underlying the analgesic effect of PE out of CP context (see above). The experimental cold-induced pain was preferred as the closest and most reliable experimental model mimicking clinical CP, owing to its tonic nature and engaged physiological mechanisms (Rainville et al., 1992; Gram et al., 2015; Hansen et al., 2017; Terrighena et al., 2017).

Despite controversial reports on the effect of intensive physical training (Monda et al., 2017; El-Sayes et al., 2020; Nicolini et al., 2021; Hendy et al., 2022), we expected an increase in the GABAergic input, based on the results obtained in EIH settings (Gramkow et al., 2020) (see above). Furthermore, we assumed that individuals with more intensive PE (e.g., endurance sports) would have a better response to cold-induced pain. We also made the assumption that some premorbid differences in indicators of central sensitization and other CP-related aberrant behaviors, could be associated to differences in pain response between the two training groups (Lentini et al., 2021). These differences would possibly feature responsiveness to exercise-induced analgesia or susceptibility to develop CP. Moreover, we assumed that these differences would be more prominent between highly trained and non-trained individuals.

## 2 Materials and methods

### 2.1 Study type and ethics

This monocentric case–control study submitted healthy highly trained athletes and age-adjusted non-trained controls to experimental pain induced by cold stimulation. The study fulfilled all the requirements of the Declaration of Helsinki and was approved by the Ethical Committee of Vaud under the reference 2019-00442. Each participant signed an informed consent prior to any investigation and received a financial compensation thereafter as requested by the Ethical Committee.

### 2.2 Participants

Participants were recruited mainly through public advertisements, social networks and clubs dedicated to endurance sports in the Canton of Fribourg (Switzerland). The primary eligibility criteria consisted in adult age (≥18 years), right-handed reported manual laterality (because of potential lateralization in pain-related brain function; Teixeira et al., 2021) and male sex (to mitigate potential data variability due to sex-based differences in pain perception; Fillingim, 2000; Kowalczyk et al., 2006). The decision to exclude women was therefore neither arbitrary nor discriminatory in this exploratory study aiming to detect group differences with the least bias as possible. The group of athletes was defined by ≥7 h of training per week in endurance sports (e.g., triathlon, running, or cycling) during the last 6 months before recruitment, whereas controls were engaged in PE or sports (to be distinguished from physical activity; Nicolini et al., 2021) for <2.5 h per week during the last 6 months before the experiments. Exclusion criteria were the presence of any type of pain, known or documented neurological dysfunction or lesions, severe sleep disorders (that could interfere with EEG results), significant cognitive or psychiatric disorders (which could prevent from appropriate evaluation), and limb ischemia, diabetes, respiratory and cardiovascular disease, Raynaud’s phenomenon, or musculoskeletal lesions or diseases (in order to avoid symptom aggravation or complications due to cold stimulation; Pienimäki, 2002).

### 2.3 Data collection

#### 2.3.1 General data

All recruited participants were individually submitted to a formal questionnaire to collect their personal and medical information, as well as details regarding their physical (sport) activity and sleep habits. The Insomnia Severity Index (ISI) (Omachi, 2011) was compiled to assess sleep difficulties, while the Hospital Anxiety and Depression (HAD) (Zigmond and Snaith, 1983) scale evaluated mood disorders (anxiety and depression more specifically).

#### 2.3.2 The cold pressure test

The cold pressor test (CPT) was the procedure used to induce pain and determine pain threshold and tolerance. It was conducted at room temperature (at ~20°C) using two different Plexiglas trays (36 cm×23.5 cm×26 cm) further isolated with an external 3 cm-thick layer of ROOFMAT polystyrene to maintain a stable temperature as long as possible. The first tray was half-filled with warm water (water volume estimated to 8 L) from the tap (~32°C). The second tray was also half-filled, but with cold water from the tap (~4 L) and with crushed ice from a small container (~4 L volume). This procedure was efficient enough to maintain the cold-water temperature around the desired level of ~4°C, as shown through continuous monitoring (mean ± SD: 4.18 ± 0.49°C) using an electronic immersible thermometer (Traceble Excursion-trac Datalogging Thermometer). That way, we could avoid a variation of ≥2°C that could affect experimental results (Mitchell et al., 2004).

Prior to the experiment, the participant was allowed to briefly immerse his non-dominant (left) hand in the iced-cold water to feel the experimental temperature and avoid unwanted anxiety that could affect his response. Right before and after the CPT, the participant immersed his right-hand in warm water at 32°C (mean ± SD: 31.8 ± 0.92°C) during 2 min for procedure standardization before cold-induced pain and for his comfort during recovery (see below).

**Figure 1:**
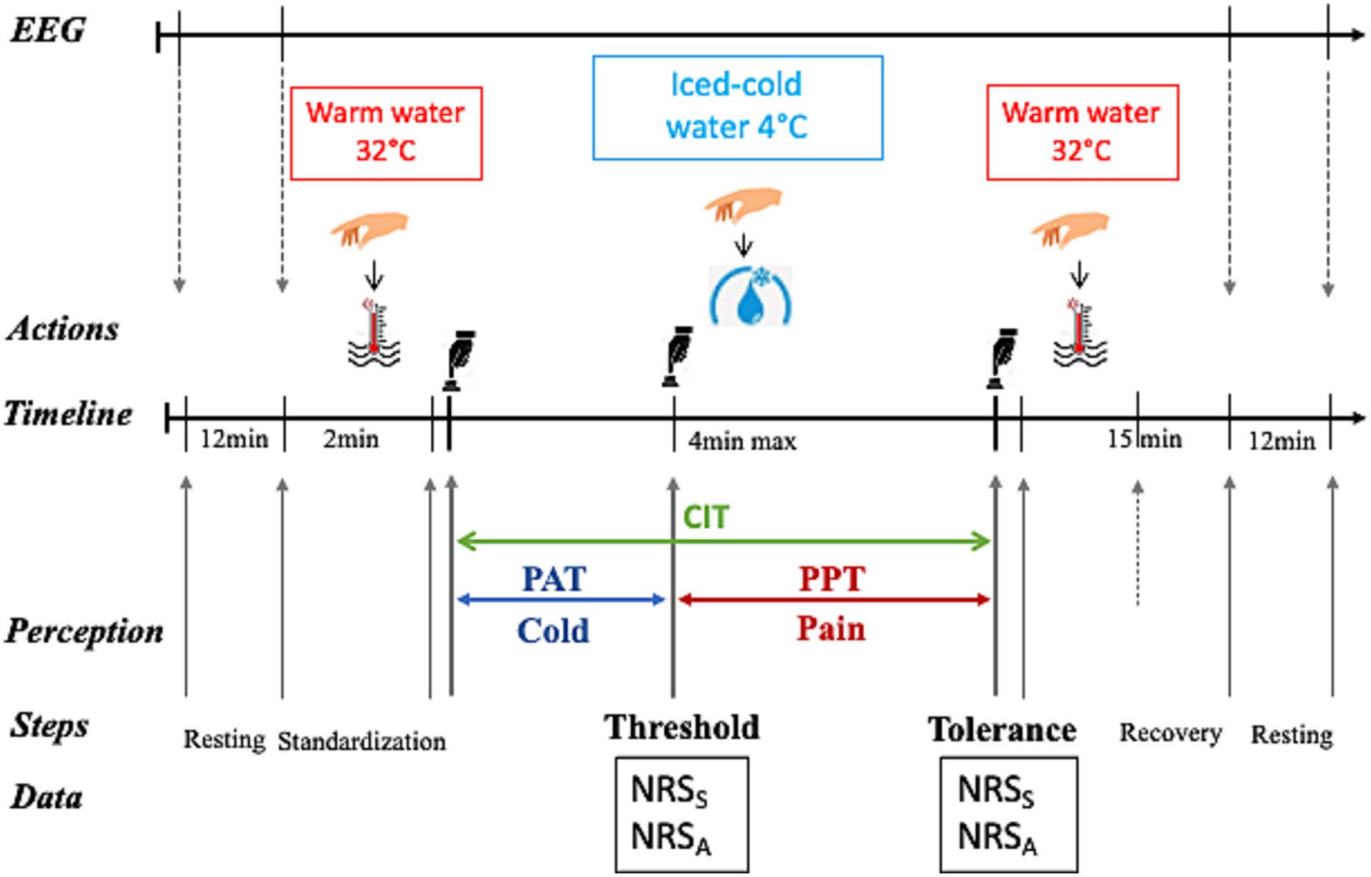
The cold pressure test (CPT) procedure. A continuous EEG recording was performed during the whole procedure (black right-oriented arrow on top of the figure), as well as an additional resting-state recording before and after the CPT. After 2 min of warm water immersion at 32°C (red thermometer in water), the participant immersed his right hand in iced cold-water at 4°C (blue water drop with ice) for a maximal duration of 4 min and thereafter again in warm water to recover for 15 min. The time elapsed between cold water immersion and the appearance of pain (pain threshold, THR) corresponded to the pain appearance time (PAT, blue horizontal line with double arrow), whereas the time between pain appearance and the maximal pain (pain tolerance, TOL; when the participant was required to remove his hand from the cold water) represented the pain perception time (PPT, blue horizontal double arrowed line). The cold immersion time (CIT, green double arrow) was the sum of PAT and PPT. The respective levels of pain intensity (indicating the sensory pain, S) and unpleasantness (assessing the affective pain, A) were separately measured using the numerical rating scale (NRS) at THR and at TOL. Key experimental step timing was recorded using E-Prime 3.0-generated triggers initiated by the investigator (black hand above the button) upon the participant’s indications. The illustrating cartoons (Right hand, Hot water, Cold water and Black hand pushing the button) were dowloaded as freely available images from the web links in September 2021.

The CPT procedure (illustrated in Figure 1) properly started as the participant immersed his right hand in cold water up to his wrist. His hand had to be positioned horizontally and relaxed in water without touching the bottom of the tray. To avoid water warming up around the hand, the participant had to gently and slowly move his hand while keeping it in a horizontal position. He was instructed to inform the investigator as soon as he began to experience pain. At that moment, he had to verbally rate the intensity and the unpleasantness level of the perceived pain, giving two separate numbers out of 10 on a scale from 0 to 10 using the numeric rating scale (NRS) displayed in front of him on the wall. This exact experimental step (i.e., the switch from cold to pain perception) represented the participant’s pain threshold (THR). As the experiment evolved, pain level increased, and the participant was required to remove his hand from the cold water when pain became “unbearable.” After cold-water removal, he immersed his hand in warm water for his comfort and to recover from pain sensation. This maximal pain time-point represented the participant’s pain tolerance (TOL). Again, as for the THR, the participant was required to quantify his pain intensity and unpleasantness levels at that moment. The maximal cold-immersion time allowed was 4 min, in order to prevent and limit the risk of tissue damage (MacLachlan et al., 2016). However, the participant was left blind to this predetermined maximal duration to avoid targeting. Each experimental step (start, cold-water hand immersion, pain appearance, hand removal time from cold water, end) was precisely recorded using the E-Prime 3.0 program (Psychology Tools, Inc., Pittsburgh, PA, USA) though triggers controlled by the investigator upon the participant’s indications.

Response to pain stimulation was documented and interpreted as following. The level of pain intensity and unpleasantness (separately quantified using the NRS, see above) assessed, respectively, the participant’s sensory and affective dimensions of pain. The latter represents the emotional experience associated to- and in response to pain (Melzack, 1975, 1987). The THR was indicated by pain intensity and unpleasantness at the moment when cold perception switched to pain perception, as well as by the time elapsed between cold-water immersion and appearance of pain (referred to as pain appearance time). Pain intensity and unpleasantness at THR and pain appearance time were considered as indicators of the sensitivity to pain stimulation (or pain sensitivity). The pain perception time was defined as the time duration between pain onset and hand removal from cold water and represented an estimate of resistance to pain (or pain resistance). The sum of pain appearance time and pain perception time represented the cold immersion time.

#### 2.3.3 Pain-related behavioral data

Although this study exclusively investigated pain-free persons, we were interested, as stated above, in detecting some premorbid traits in indicators of pain-related behaviors, which would expectedly influence response to experimental pain. Conceptually, they would constitute premises of- or indicate some susceptibility to develop maladaptive behaviors toward pain in CP contexts. In addition to central sensitization (largely discussed above), catastrophizing beliefs and fear of pain were targeted, knowing also that they can affect experimental pain experience (Sullivan et al., 2001; George et al., 2006), especially in highly trained individuals (Lentini et al., 2021). In absence of validated tools to assess these behavioral features in pain-free populations, we used, respectively, the Central Sensitization Index (CSI) (Neblett et al., 2013), the Pain Catastrophizing Scale (PCS) (Sullivan et al., 2001; Lentini et al., 2021) and the Fear of Pain Questionnaire (FPQ-9) (McNeil et al., 2018). Participants were submitted to these standard questionnaires on the experimental day, prior to the CPT procedure.

#### 2.3.4 Electroencephalographic recordings

Brain GABAergic activity can be reliably measured by electroencephalography (EEG) (Barr et al., 2013), through fast oscillations (β (13–30 Hz) and ɤ (30–100 Hz) waves); the latter being mainly driven by inhibitory interneurons (Gaetz et al., 2011; Christian et al., 2015; Baumgarten et al., 2016). In this study, we analyzed β EEG activity, which is more reliably measured by scalp EEG than deeply generated ɤ activity (Teixeira et al., 2021) and is related to GABA levels in the somatosensory cortex (Baumgarten et al., 2016; Teixeira et al., 2021).

Thus, a continuous high-density (64 electrodes) resting-state EEG recording (BIOSEMI Active Two recording system) was conducted at baseline before and after the whole experiment in three randomized conditions lasting 3 min each: seated with eyes open, seated with eyes closed and standing with eyes closed (see Figure 1). In addition, the EEG recording took place throughout the experiment, without other tasks than instructions given to participants (e.g., hand immersion, hand removal, etc). The EEG recording was performed at a sampling rate of 1,024 Hz, referenced to the common mode sense-driven right leg (CMSDRL) ground placed on each side of the POz electrode.

### 2.4 Data analysis

#### 2.4.1 Electroencephalography preprocessing and spectral analysis

Automated EEG data preprocessing was performed offline, using a customized MATLAB toolbox (EEGpalCS), including functions from the EEGLab Toolbox (Delorme et al., 2011). First, raw EEG data were bandpass filtered by a high pass of 1 Hz and a low pass of 60 Hz using Finite Impulse Response (FIR) filters (Winkler et al., 2015). Automated artefacts rejection algorithms (EEGlab plugin) were applied to remove sinusoidal noise stemming from alternating current powerline fluctuation [CleanLine (Mullen, 2012)]^1^ or high amplitude eye movements, muscle artefacts, and electrode drifts (ASR; Mullen et al., 2015; Chang et al., 2018). Bad EEG channels were excluded by visual inspection using the Cartool software (Brunet et al., 2011) for data visualization (limited to a maximum of seven rejected electrodes). Then, selected channels were interpolated using spherical splines (Perrin et al., 1989) [median (IQR) = 3.1 (24.7) % interpolated electrodes]. Obtained preprocessed data were re-referenced to the average of all electrodes and segmented in 1-s epochs using EEGpalCS. All epochs containing one channel or more above the threshold were automatically rejected from the final analysis to eliminate remaining artefacts. This study set the artefactual signals threshold to 100 μV according to our laboratory standard.

Finally, retained epochs were recomputed into the frequency domains using the Fast Fourier Transform (FFT) with a frequency resolution of 1 Hz. The β frequency domain (13–30 Hz) constituted the main focus of interest in this investigation (see above). In addition, in this exploratory study, we also analyzed the δ (2–4 Hz) and α (7–12 Hz) frequency ranges to increase the specificity of our observations regarding GABAergic markers (Pinheiro et al., 2016; Mussigmann et al., 2022). The spectral power of each frequency was divided by the average power of all epochs for that given frequency to remove 1/f noise. Frequency bands were obtained by averaging all frequency bins within, respectively, each frequency range. The global power spectrum (GPS, i.e., the frequency power within the band averaged across all electrodes) constituted the quantitative measure of the power for a given frequency band.

The β frequency domain was previously shown to have two power peaks in chronic neuropathic pain patients (Teixeira et al., 2021), yielding two sub-bands; namely the high (20–30 Hz) and the low (13–20 Hz) β ranges (respectively abbreviated as Hβ and Lβ). Because this study was conducted in relation with CP and using the same analysis paradigm, we similarly discriminated the two β sub-bands in our analyses.

In order to evaluate the individual GPS corresponding to different sensory perceptions (i.e., warm, cold or pain) during the experiment, the GPS of each participant was averaged, respectively, over the total duration of each perception in each frequency domain. For each frequency domain, the participant’s GPS at threshold was computed as the mean value within the time interval between 2 s before and 2 s after the threshold, considering that there could be a small error between the real pain appearance time and when the participant indicated pain appearance to the experimenter. At TOL, because the movement of hand removal from the cold water induced an important EEG artefact, we considered the mean GPS during the 4 s of clean recording closest to the hand removal time.

#### 2.4.2 Statistical analyses and power estimation

Statistical analyses were conducted using the Jamovi Statistical Software (version 2.2.5; Sydney, Australia) and RStudio [Version 2023.06.1 + 524 (2023.06.1 + 524)]. Clinical data were all quantified as mean ± SD and analyzed with parametric statistical tests for easiness of interpretation, although some of them were not normally distributed. In contrast, the CPT-related psychophysical data and all EEG data displayed non-normal distribution and were thus presented as median (IQR) and analyzed using non-parametric tests.

The significance threshold was set at *p* < 0.05 (two-tailed; 95% CI). Differences in clinical data between groups were assessed by an independent sample *t*-test (simple comparison between groups) or multivariate analysis of variance (ANOVA; when comparison between groups implied more than one condition). Differences in CPT psychophysical data were assessed using the Welch’s t-test, while differences within and between groups in EEG data were assessed using repeated measures multivariate ANOVA. The Tukey post-hoc analysis corrected for multiple comparisons. In this exploratory study, in order to complete data interpretation beyond statistical significance, the effect size was further computed in most comparative analyses using the Cohen’s *d* [small effect if *d* ~ 0.2, moderate effect if *d* ~ 0.5 and large effect if *d* ≥ 0.8; (Lakens, 2013)]. In order to refine the analysis of EEG data, we also computed differences between different perceptions (normalized as % to the value at pain perception) and between THR and TOL values (normalized as % to the TOL value). Correlations were assessed employing the Spearman Rho (r_s_) correlation coefficient (two-tailed) and correction for multiple testing performed by the Benjamin Holm (BH) procedure, while the effect size was evaluated as follows: small correlation if −0.5 < *r*_s_ ≤ 0 or 0 ≤ *r*_s_ < 0.5, large correlation if −1.0 < *r*_s_ ≤ −0.5 or 0.5 ≤ *r*_s_ < 1.0 (Akoglu, 2018).

When significance vanished upon multiple testing corrections in above-mentioned inferential analyses, only results with large size effect were mentioned, and considered for further discussion in coherence with persistently significant data.

It was challenging to precisely perform an *a priori* sample size estimation in such an exploratory study investigating both clinical and EEG data. Indeed, while one pain study with similar design (between and within comparisons, correlations), assessing clinical and psychophysical parameters (e.g., pain intensity and unpleasantness) related to exercise-induced hypoalgesia, calculated a total sample size of 26 (13 per group) for moderate to high power (Cohen’s *d* of 0.5, two-tailed alpha of 0.05 and a power of 0.8) (Peterson et al., 2019), a longitudinal study of electrophysiological indicators of exercise-induced cortical excitability yielded a sample of 22 participants for statistical similar power (Lulic et al., 2017; El-Sayes et al., 2020).

## 3 Results

### 3.1 General demographic, clinical and EEG characteristics

**Figure 2:**
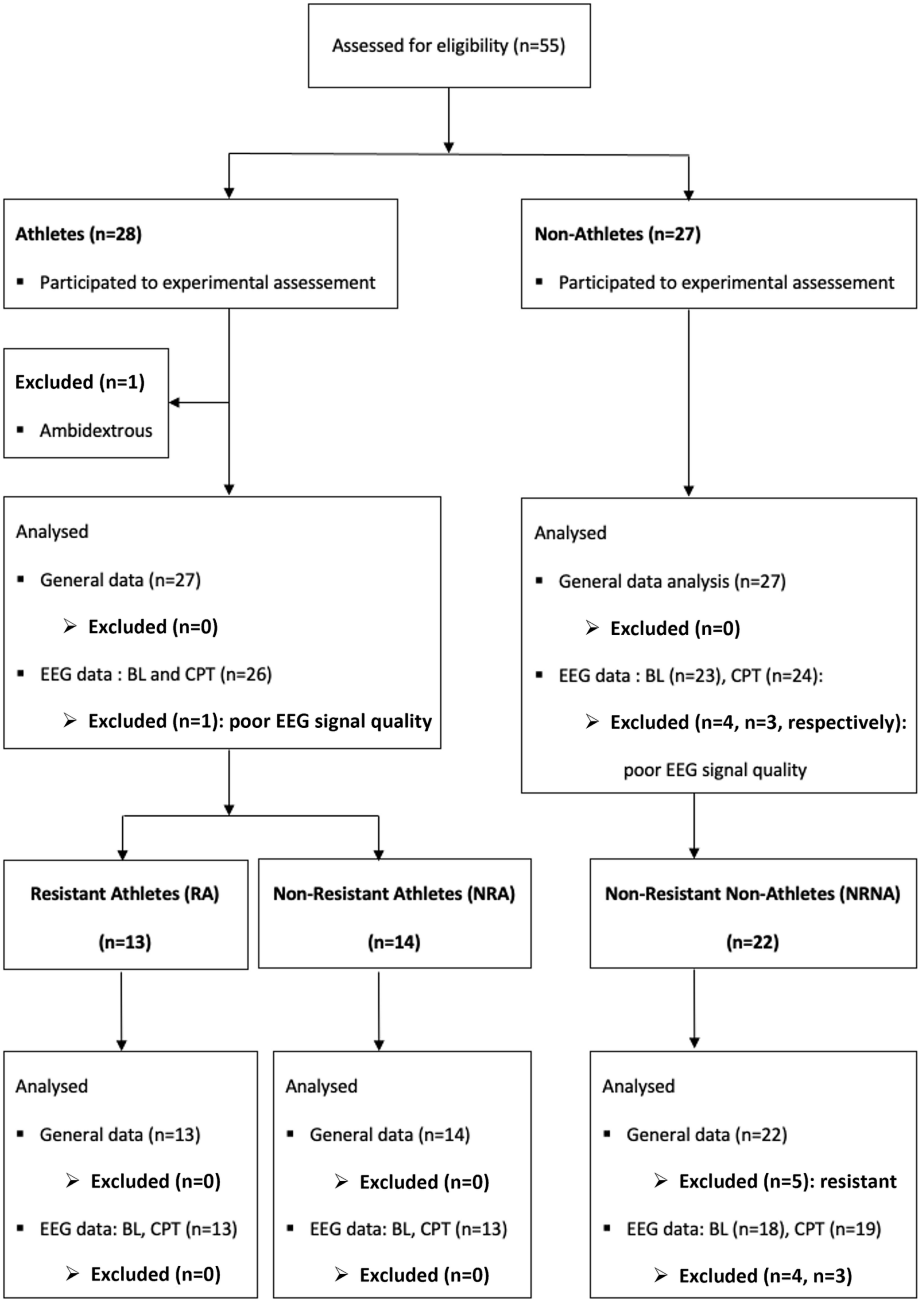
Overview of the selection procedure for participants and data at different analysis steps. In total 55 participants were screened (28 athletes and 27 non-athletes). One athlete was secondarily excluded from the primarily analysis because he was ambidextrous. In addition, 4 participants (1 athlete and 3 non-athletes) were excluded from the EEG analysis and one more non-athletes from the EEG analysis at baseline (BL) because of the poor quality their recordings. For the specific analysis of the three categories related to pain resistance (RA, NRA and NRNA), 5 resistant non-athletes were excluded because of the small sample and the same participants excluded from the EEG data analysis belonged, respectively, to the non-resistant athlete (NRA, *n* = 1) and to the non-athletes [NA, baseline (BL): *n* = 4, cold pressure test (CPT): *n* = 3] groups.

In total, 55 participants were screened and enrolled, among which one athlete was secondarily excluded as found to be ambidextrous (Figure 2). The primary analysis was thus performed on 27 athletes and 27 non-athletes (mean ± SD age 35.4 ± 8.43 years and 41.6 ± 10.2 respectively). Further specific comparative analyses were conducted between two sub-groups of 13 and 19 participants (see below for more information).

An overview of general demographic and clinical characteristics of the participants can be found in Table 1 (top part). Athletes devoted nearly ten times more hours per week to their training compared to non-athletes (*p* < 0.001, large effect size), were significantly older (*p* = 0.019, moderate effect size) and had a lower body mass index (BMI) (*p* < 0.001, large effect size). The HAD_A_ and HAD_D_ subscales and ISI scores were not significantly different between the two groups and remained within normal ranges. Pain-related behavior scores (CS, FPQ-9 and PCS) were all lower in athletes, but only the FPQ-9 reached statistically significant difference (*p* = 0.006, large effect size).

**Table 1:** Participants’ demographic and general characteristics.

	A (*n* = 27)	NA (*n* = 27)	*p*	Cohen’s *d*
Age [years]	35.4 (8.43) (20–53)	41.6 (10.2) (23–58)	0.019	0.658
BMI [kg/m^2^]	22.7 (2.43) (17.9–27.1)	26.4 (3.44) (20.6–37.5)	<0.001	1.258
Hours of training [h/week]	11.4 (0.94) (8.0–20.0)	1.34 (3.21) (0.0–2.5)	<0.001	4.231
HAD_A_ [/21]	6.00 (2.66) (0–11)	6.41 (3.59) (1–18)	0.637	0.129
HAD_D_ [/21]	2.22 (1.83) (0–6)	2.89 (2.59) (0–9)	0.280	0.297
ISI [/28]	7.59 (5.23) (0–18)	7.04 (5.35) (0–18)	0.701	0.105
Sleep duration [h/night]	7.97 (0.95) (6.0–9.50)	7.69 (0.66) (6.0–9.0)	0.203	0.350
CSI [/100]	19.9 (8.74) (1–35)	22.0 (11.3) (3–49)	0.446	0.209
FPQ-9 [9 to 45]	18.6 (3.86) (10–27)	22.4 (5.60) (9.0–34)	0.006	0.785
PCS [/52]	9.56 (4.71) (2–19)	11.6 (8.67) (1–28)	0.280	0.297

	RA (*n* = 13)	NRNA (*n* = 22)	*p*	Cohen’s *d*
Age [Years]	35.2 (9.54) (20–53)	41.4 (10.8) (23–58)	0.099	0.595
BMI [kg/m^2^]	22.5 (2.03) (19.6–26.8)	26.1 (2.84) (20.6–33.2)	<0.001	1.401
Hours of training [h/week]	11.8 (2.67) (8–15)	1.26 (1.5) (0–2.5)	<0.001	5.970
HAD_A_ [/21]	5.85 (2.82) (2–11)	6.64 (3.62) (2–18)	0.505	0.236
HAD_D_ [/21]	2.15 (1.68) (0–6)	3.27 (2.68) (0–9)	0.185	0.474
ISI [/28]	6.54 (4.91) (0–16)	7.14 (5.24) (0–18)	0.741	0.117
CSI [/100]	19.0 (7.43) (6–31)	23.1 (11.5) (3–49)	0.261	0.400
FPQ-9 [9 to 45]	18.8 (2.51) (13–23)	22.8 (5.97) (9–34)	0.030	0.795
PCS [/52]	9.54 (4.59) (2–18)	12.7 (8.58) (1–28)	0.226	0.432

Data are shown as mean (SD) and values below represent data range (min - max). The p values result from an Independent Sample Students’ t-test and displays differences between Athletes (A) and Non-Athletes (NA) (upper part), as well as between Resistant-Athletes (RA) and Non-Resistant Non-Athletes (NRNA) (lower part). Numbers in bold display statistical significance (*p* < 0.05, 95% CI). The effect size was evaluated with the Cohen’s *d* as following: small effect (*d* ~ 0.2), moderate effect (*d* ~ 0.5) and large effect (*d* > 0.8). BMI, Body Mass Index; HAD, Hospital Anxiety and Depression scale for anxiety (HAD_A_) and for depression (HAD_D_); ISI, Insomnia Severity Index; CSI, Central Sensitization Index; FPQ-9, Fear of Pain Questionnaire (9 Items); PCS, Pain Catastrophizing Scale.

**Figure 3:**
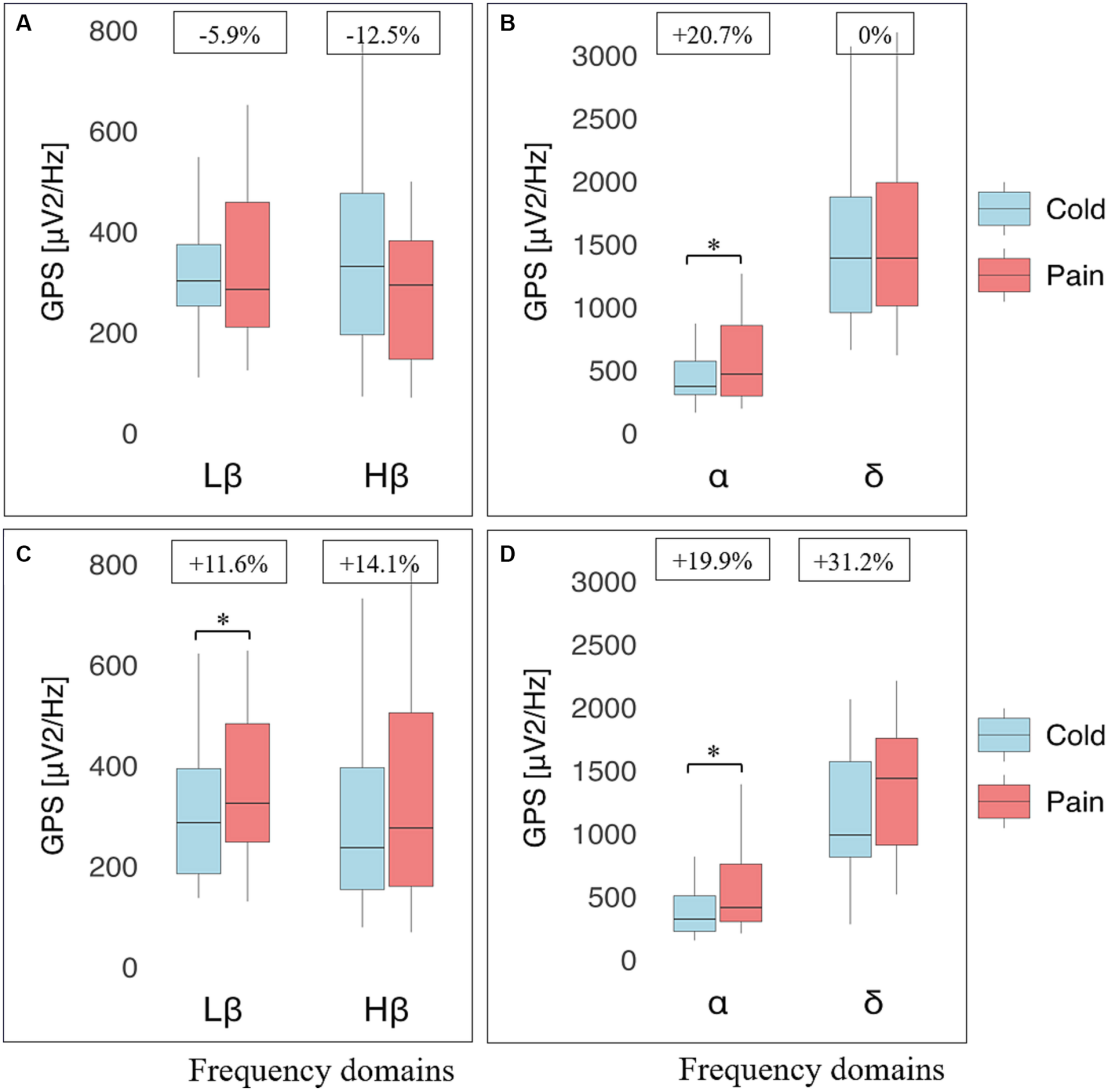
Electroencephalographic (EEG) global power spectra (GPS) according to frequency bands during cold and pain perceptions in athletes and non-athletes. GPS (in μV^2^/Hz) are presented on the y-axis as median (horizontal black line) and interquartile range (IQR, upper and lower edges of the box), while grey whiskers indicate minimum and maximum values. GPS during cold and pain perceptions are, respectively, shown in light blue and light red colors. Different frequency bands are represented on the *x*-axis: Low Beta (Lβ; 13-20 Hz) and High Beta (Hβ; 20–30 Hz) in A and C graphs; Alpha (α; 8–12 Hz) and Delta (δ; 2–4 Hz) in B and D graphs. The numbers at the top indicate GPS decrease (−) or increase (+) between cold and pain in percentage (%) normalized to the value during pain perception. Athletes (top panels) showed a decrease in GABAergic markers **(A)**; respectively from 302 (122) to 285 (248) μV^2^/Hz (Lβ, *p* = 1.0) and from 331 (281) to 294 (235) μV^2^/Hz (Hβ, *p* = 0.318). In contrast, an increase was observed in the *α* band (from 371 (266) to 468 (560) μV^2^/Hz; *p* = 0.018) whereas δ GPS remained unchanged (from 1390 (918) to 1,390 (979); *p* = 0.912) **(B)**. Non-athletes (bottom panels) displayed a systematic increase in all frequency bands: Lβ (from 287 (209) to 325 (235) μV^2^/Hz; *p* = 0.014) and Hβ (from 237 (242) to 276 (344) μV^2^/Hz; *p* = 0.903) **(C)**; α (from 317 (281) to 396 (335) μV^2^/Hz; *p* = 0.010) and δ (from 989 (756) to 1,438 (847); *p* = 0.059) **(D)**. *Indicates significant results (*p* < 0.05, 95% CI) from a repeated ANOVA and a Tukey tested for post-hoc differences, while at the same time correcting for multiple testing.

Four participants were excluded from EEG analyses because of poor data quality during the pre-processing step (Figure 2). The remaining 26 athletes and 24 non-athletes were comparatively analyzed, focusing on data collected in standing position with eyes closed at baseline (i.e., before hand immersion) and during warm, cold and pain perceptions. Our analysis indicates no significant difference between the two groups at any frequency, at baseline and during all perceptions (data not shown). In contrast, a significant interaction was noticed in the Lβ frequency band (*p* = 0.026, small effect size) between cold and pain perceptions when comparing the two groups, with non-significant post-hoc Lβ and Hβ GPS *decrease*, *unchange*d δ GPS, but a significant α GPS *increase* (*p* = 0.038, small effect size) between perceptions in athletes. In non-athletes, there was a power *increase* at all frequencies, reaching statistical significance within the Lβ (*p* = 0.030, small effect size) and the *α* (*p* = 0.022, moderate effect size) ranges (Figure 3).

### 3.2 The unique profile of resistant athletes

During the CPT, a category of participants did not experience unbearable pain within the maximal limit of immersion time (4 min). They were qualified as “resistant” for this reason and were requested to remove their hands from the cold water before experiencing maximal pain for safety reasons, according to the protocol (see methods). Interestingly, they were more commonly represented among athletes (48.1%; *n* = 13) than among non-athletes (18.5%; *n* = 5). Because the number of “resistant” non-athletes was too small, they were excluded in the remainder analyses. In order to take into consideration the potential ability of resistant athletes (RA) to exceed the artificially settled maximal immersion time (which probably introduced a biasing “ceiling effect”), we considered their clinical and EEG data separately from those of non-resistant athletes (NRA) and of non-resistant non-athletes (NRNA) (Figure 2).

According to primary analyses, the most significant differences were noticed between RA and NRNA, which oriented the focus of subsequent analyses on them (data from NRA are shown in the Supplementary material).

### 3.3 Comparative analysis of RA and NRNA profiles

The new comparative analysis between RA (*n* = 13) and NRNA (*n* = 22) found similar trends as for the general comparison between athletes and non-athletes (see above) regarding all clinical parameters, except age (which, despite similar results, was now non-significantly different) (Table 1, bottom part).

**Figure 4:**
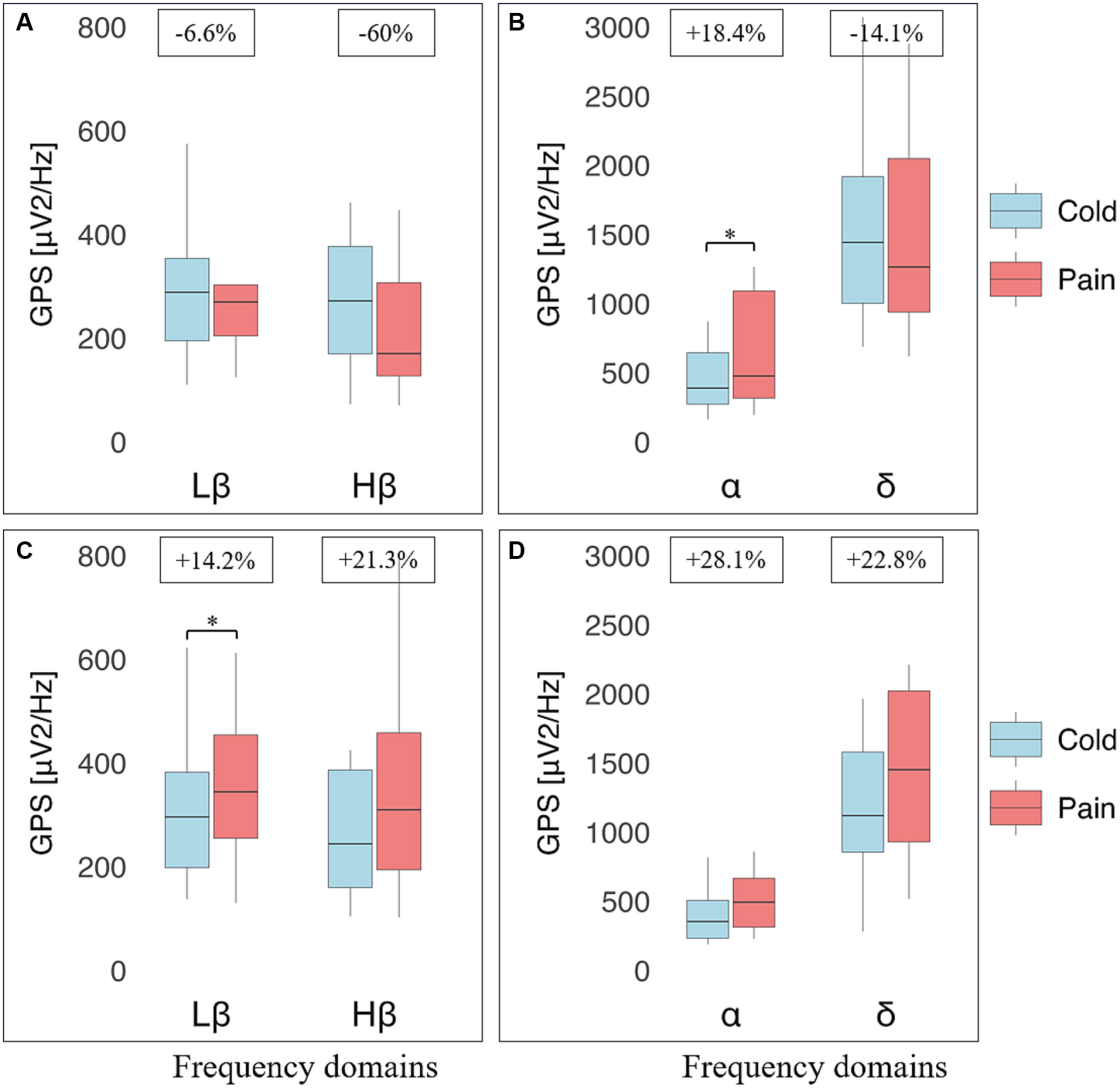
Electroencephalographic (EEG) global power spectra (GPS) according to frequency bands during cold and pain perceptions in resistant athletes (RA) and non-resistant non-athletes (NRNA). GPS (in μV^2^/Hz) are presented on the y-axis as median (horizontal black line) and interquartile range (IQR, upper and lower edges of the box), while grey whiskers indicate minimum and maximum values. GPS during cold and pain perceptions are, respectively, shown in light blue and light red colors. Different frequency bands are represented on the x-axis: Low Beta (Lβ; 13–20 Hz) and High Beta (Hβ; 20–30 Hz) in A and C graphs; Alpha (α; 8–12 Hz) and Delta (δ; 2–4 Hz) in B and D graphs. The numbers at the top indicate GPS decrease (−) or increase (+) between cold and pain in percentage (%) normalized to the value during pain perception. RA (top panels) showed a decrease in GABAergic markers **(A)**; respectively from 288 (159) to 270 (98.1) μV^2^/Hz, (Lβ, *p* = 0.975) and from 275 (207) to 170 (180) μV^2^/Hz (Hβ, *p* = 0.183), as well as δ GPS (from 1443 (915) to 1264 (1109) μV^2^/Hz; *p* = 0.874). In contrast, an increase was observed in the α band (from 389 (371) to 477 (774) μV^2^/Hz; *p* = 0.045) **(B)**. NRNA (bottom panels) displayed an increase in all frequency bands: Lβ (from 296 (184) to 345 (199) μV^2^/Hz; *p* = 0.030) and Hβ (from 244 (226) to 310 (264) μV^2^/Hz; *p* = 0.734) **(C)**; α (from 355 (274) to 494 (354) μV^2^/Hz; *p* = 0.072) and δ (from 1120 (722) to 1452 (1091) μV^2^/Hz; *p* = 0.057) **(D)**. *Indicates significant results (*p* < 0.05, 95% CI) from a repeated ANOVA and a Tukey tested for post-hoc differences, while at the same time correcting for multiple testing.

At baseline, there was again no statistically significant differences in EEG markers (eyes closed, standing position) at all perceptions between RA and NRNA (see Supplementary Table 1). However, significant interactions were found for Hβ (*p* = 0.031, small effect size) and Lβ (*p* = 0.026, small effect size) GPS, when comparing the two groups during cold and pain perceptions (Table 2, top part), with no significant post-hoc *decrease* in RA and *increase* in NRNA Hβ GPS, but significant *increase* in Lβ GPS for NRNA (*p* = 0.030, small effect size) and non-significant *decrease* for RA (Figure 4). An interaction was also noticed in the δ domain (non-significant post-hoc differences), but not in the α band (where, though, a significant *increase* was observed in RA between cold and pain perception; *p* = 0.045, moderate effect size) (Figure 4). It should be finally noted that there existed a “within” effect related to the experimental step (i.e., from cold to pain perception) in the α frequency range (Table 2, top part).

**Table 2:** Results from repeated ANOVA between RA and NRNA across frequency ranges.

Dependent measures	COLD VS PAIN PERCEPTIONS
Hß GPS	PERCEPTION_(1,30)_ = 0.894; *p* = 0.352
	GROUP_(1,30)_ = 0.245; *p* = 0.624
	**PERCEPTION × GROUP**_**(1,30)**_ **= 5.097; *p* = 0.031**
Lß GPS	PERCEPTION_(1,30)_ = 2.87; *p* = 0.101
	GROUP_(1,30)_ = 0.354; *p* = 0.556
	**PERCEPTION × GROUP**_**(1,30)**_ **= 5.45; *p* = 0.026**
α GPS	**PERCEPTION**_**(1,30)**_ **= 14.116; p < 0.001**
	GROUP_(1,30)_ = 0.683; *p* = 0.415
	PERCEPTION × GROUP_(1,30)_ = 0.257; *p* = 0.616
δ GPS	PERCEPTION_(1,30)_ = 1.25; *p* = 0.273
	GROUP_(1,30)_ = 0.00583; *p* = 0.940
	**PERCEPTION × GROUP**_**(1,30)**_ **= 5.19; p = 0.030**

Dependent measures	THRESHOLD VS TOLERANCE TIME POINTS
Hß GPS	TIME POINTS_(1,30)_ = 3.07; *p* = 0.09
	GROUP_(1,30)_ = 1.05; *p* = 0.314
	**TIME POINTS × GROUP**_**(1,30)**_ **= 6.84; p = 0.014**
Lß GPS	TIME POINTS _(1,30)_ = 0.605; *p* = 0.443
	GROUP_(1,30)_ = 0.765; *p* = 0.389
	**TIME POINTS × GROUP**_**(1,30)**_ **= 6.621; *p* = 0.015**
α GPS	**TIME POINTS**_**(1,30)**_ **= 5.94; *p* = 0.021**
	GROUP_(1,30)_ = 0.421; *p* = 0.522
	TIME POINTS x GROUP_(1,30)_ = 1.44; *p* = 0.239
δ GPS	TIME POINTS _(1,30)_ = 2.66; *p* = 0.113
	GROUP_(1,30)_ = 0.250; *p* = 0.621
	TIME POINTS x GROUP_(1,30)_ = 0.0229; *p* = 0.881

**Figure 5:**
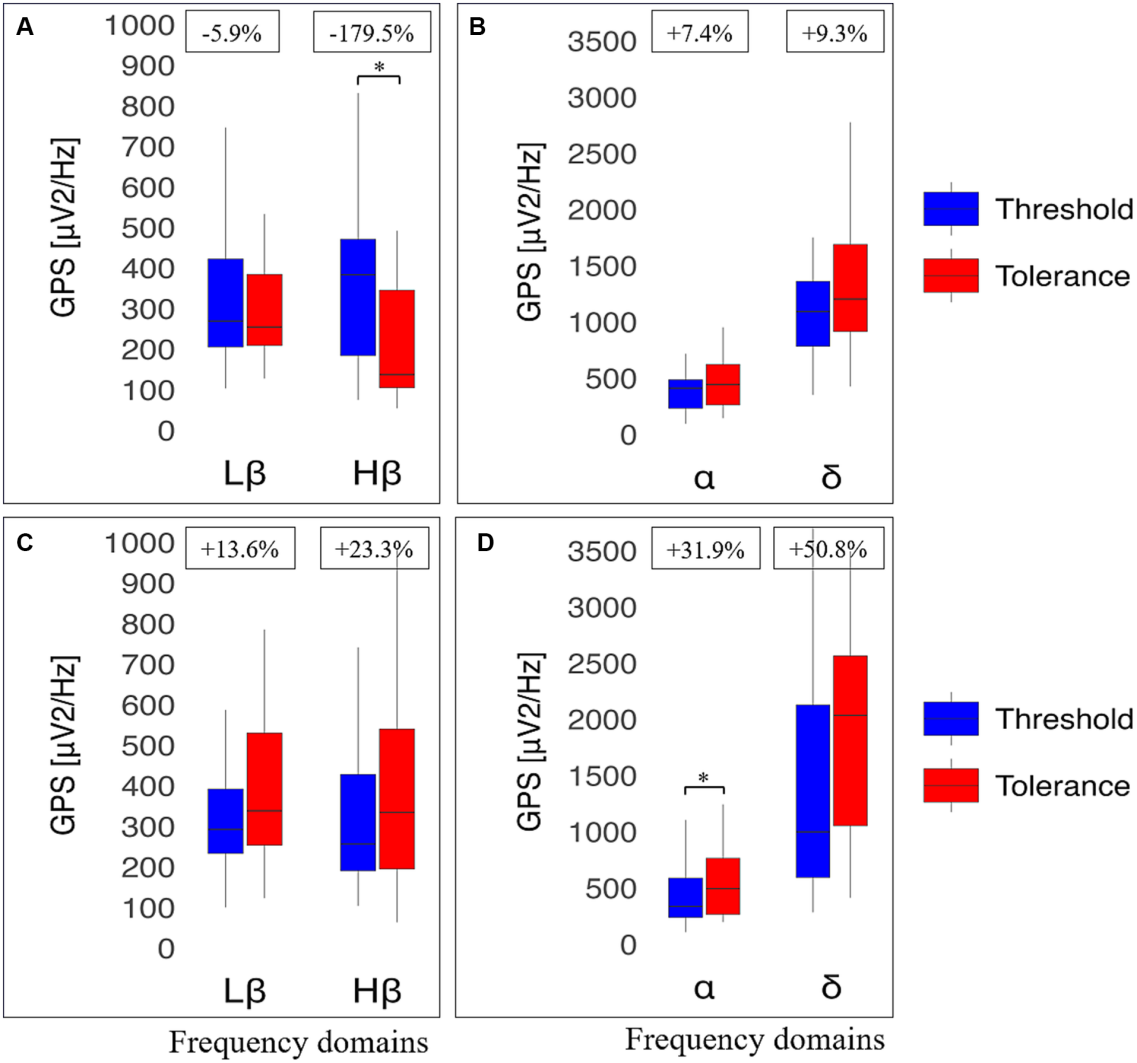
Electroencephalographic (EEG) global power spectra (GPS) according to frequency bands at pain threshold (THR) and at pain tolerance (TOL) in resistant athletes (RA) and non-resistant non-athletes (NRNA). GPS (in μV^2^/Hz) are presented on the y-axis as median (horizontal black line) and interquartile range (IQR, upper and lower edges of the box), while grey whiskers indicate minimum and maximum values. GPS at THR and TOL are, respectively, shown in bright blue and bright red colors. Different frequency bands are represented on the x-axis: Low Beta (Lβ; 13-20 Hz) and High Beta (Hβ; 20-30 Hz) in A and C graphs; Alpha (α; 8–12 Hz) and Delta (δ; 2–4 Hz) in B and D graphs. The numbers at the top indicate GPS decrease (−) or increase (+) between cold and pain in percentage (%) normalized to the value during at TOL. RA (top panels) showed a decrease in GABAergic markers **(A)**; respectively from 269 (217) to 254 (176), μV^2^/Hz (Lβ, *p* = 0.653) and from 383 (287) to 137 (240) μV^2^/Hz (Hβ, *p* = 0.039). In contrast, an increase was observed in the α band (from 411 (254) to 444 (361) μV^2^/Hz; *p* = 0.853) and in δ GPS (from 1091 (577) to 1203 (775) μV^2^/Hz; *p* = 0.658) **(B)**. NRNA (bottom panels) displayed an increase in all frequency bands: Lβ (from 292 (159) to 338 (276) μV^2^/Hz; *p* = 0.061) and Hβ (from 256 (238) to 334 (345) μV^2^/Hz; *p* = 0.905) **(C)**; α (from 336 (350) to 494 (499) μV^2^/Hz; *p* = 0.037) and δ (from 999 (1534) to 2034 (1510) μV^2^/Hz; *p* = 0.655) **(D)**. *Indicates significant results (*p* < 0.05, 95% CI) from a repeated ANOVA and a Tukey tested for post-hoc differences, while at the same time correcting for multiple testing.

Resistant athletes (RA) and non-resistant non-athletes (NRNA) are compared according to cold and pain perceptions (upper part); and between threshold (THR) and tolerance (TOL) time points (lower part). The correction for multiple comparison was performed by a Tukey post-hoc analysis (the statistical significance of post-hoc differences is presented in Figures 4, 5 in the main manuscript). Values in bold represent significant results (*p* < 0.05, 95% CI). GPS, global power spectrum; Hβ, high beta (20–30 Hz); Lβ, low beta (13–20 Hz); *α,* alpha (8–12 Hz); *δ,* delta (2-4 Hz).

The same types of analyses were conducted between the transition from cold to pain perception (THR) and the subsequent maximal pain level (TOL) in NRNA, or the safety limit of 4 min in RA (Table 2, bottom part). At THR, NRS intensity and unpleasantness were rated at the same level in both NRNA and RA (3.0/10 and 4.0/10, respectively; Table 3). Likewise, at TOL, NRS unpleasantness level was equal (8.0/10) in both groups, while RA experienced slightly but significantly *lower* NRS intensity than NRNA (*p* = 0.023, moderate to large effect size) (Table 3). Finally, pain appearance time and pain perception time were both much *longer* in RA, the difference reaching significance however only for pain perception time (*p* < 0.001, large effect size). Comparison of EEG markers between THR and TOL in RA and NRNA showed similar trends as between cold and pain perceptions (Figure 4). Accordingly, there was an interaction for Lβ (*p* = 0.015, moderate effect size) and Hβ (*p* = 0.014, moderate effect size) GPS (Table 2, bottom part), with this time a significant *decrease* of Hβ GPS in RA (*p* = 0.039, large effect size), but still non-significant slight decrease of Lβ GPS, upon post-hoc analysis. Despite absence of interaction in other frequency ranges, a significant *increase* of α GPS (*p* = 0.037, moderate effect size) was noticed in NRNA (Figure 5). The within effect was again present in the *α* band (Table 2, bottom part).

**Table 3:** Comparative analysis of response to the cold pressor test between RA and NRNA.

Variables		RA (*n* = 13)	NRNA (*n* = 22)	*p*	Cohen’s *d*
NRS [/10] Threshold	Intensity	3.0 (2.0) (1–5)	3.0 (3.0) (0–9)	0.481	0.238
	Unpleasantness	4.0 (1.0) (1–6)	4.0 (3.0) (0–10)	0.938	0.026
NRS [/10] Tolerance	Intensity	**7.0 (1.0)** **(5–9)**	**8.0 (1.75)** **(1–10)**	**0.023**	**0.779**
	Unpleasantness	8.0 (3.0) (5–10)	8.0 (2.0) (5–10)	0.683	0.148
Pain appearance time [s]		26.2 (15.4) (8.09–146)	18.6 (16.9) (8.09–54.6)	0.257	0.454
Pain perception time [s]		**214 (15.4)** **(94–232)**	**29.2 (44.8)** **(1.29–126)**	**<0.001**	**4.555**

Data are shown as median (InterQuartile Range, IQR). The *p* values result from the independent samples Welch’s *t*-test. Values in bold indicate significant differences (*p* < 0.05, 95% CI). The Cohen’s *d* evaluated the effect size as following: small effect (*d* ~ 0.2), moderate effect (*d* ~ 0.5) and large effect (*d* > 0.8). NRS, numerical rating scale; RA, Resistant Athletes; NRNA, Non-Resistant Non-Athletes.

### 3.4 Associations between clinical indicators and EEG markers of RA and NRNA

**Figure 6:**
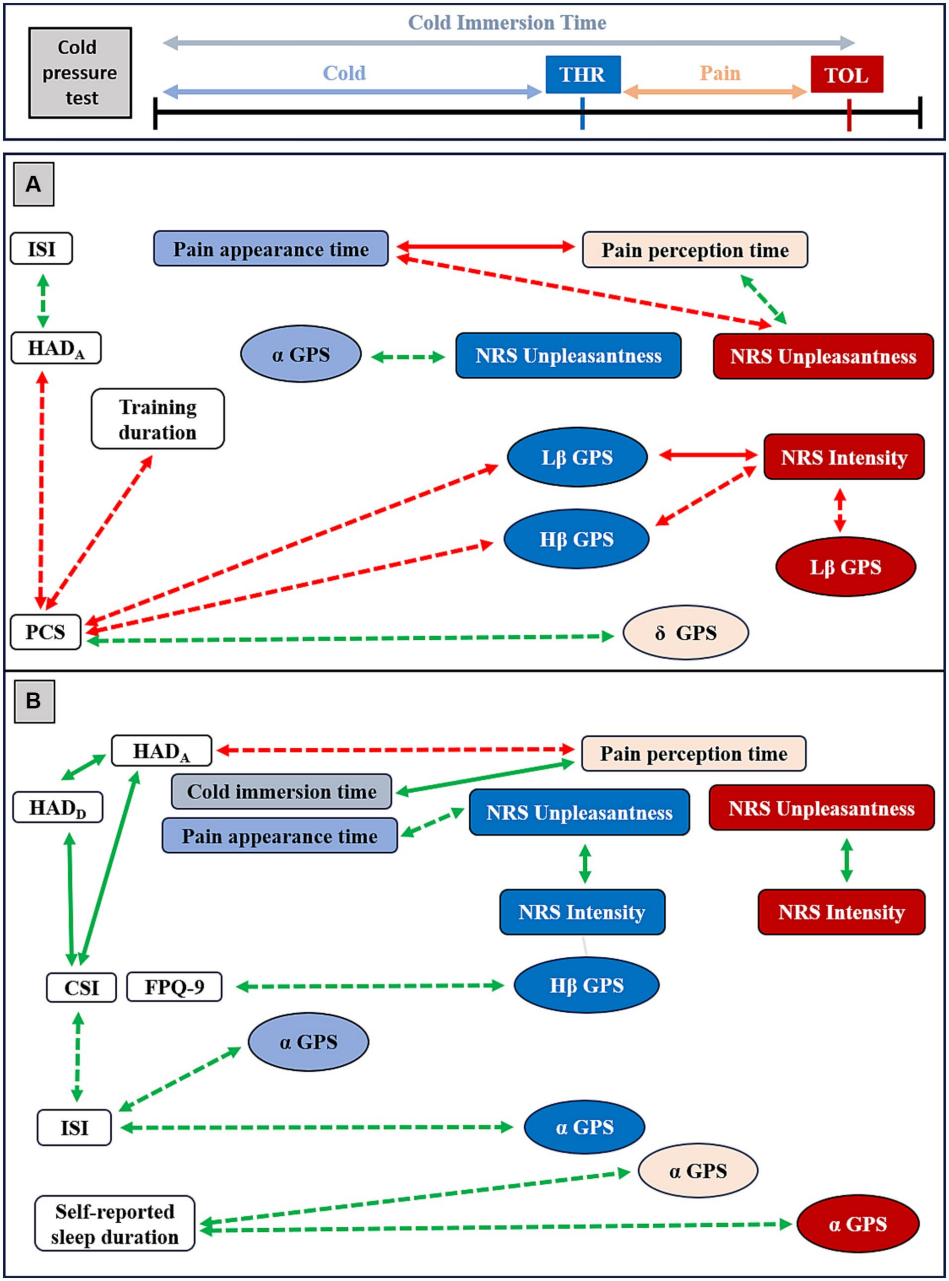
Overview of Spearman Rho (*r*_s_) correlations between clinical indicators, psychophysical data and EEG markers in resistant athletes (RA) and in non-resistant non-athletes (NRNA). Red arrows represent negative correlations and green arrows the positive ones. Plain arrows correspond to significant correlations persisting after Benjamini Hochberg (BH) correction, while dashed arrows indicate large-effect size significant correlations that disappeared upon correction. Indicators labeled in light blue and light red were evaluated, respectively, during cold and pain perceptions; those in dark blue and dark red were calculated at threshold (THR) and tolerance (TOL) time-points, respectively. HAD=Hospital Anxiety and Depression scale for anxiety (HAD_A_) and for depression (HAD_D_), PCS=Pain Catastrophizing Scale, CSI=Central Sensitization Index, NRS=Numeric Rating Scale, ISI=Insomnia Severity Index. In RA (**A**, top panel), PCS correlated to the weekly training duration (*r*_s_ = −0.569, *p* = 0.043) and to HAD_A_ (*r*_s_ = −0.563, *p* = 0.045). HAD_A_ was correlated to ISI (*r*_s_ = 0.619, *p* = 0.024). Pain appearance time and pain perception time were correlated one to each other (*r*_s_ = −1.0, *p* < 0.001) and both with NRS Unpleasantness at TOL (*r*_s_ = −0.674, *p* = 0.011 and *r*_s_ = 0.674, *p* = 0.011, respectively). In addition, Lβ GPS at THR correlated with NRS Intensity at TOL (*r*_s_ = −0.780, *p* = 0.002) and PCS (*r*_s_ = −0.637, *p* = 0.019). Lβ GPS at TOL correlated with NRS Intensity at TOL (*r*_s_ = −0.588, *p* = 0.034), Hβ GPS at THR with PCS (*r*_s_ = −0.692, *p* = 0.009) and NRS Intensity at TOL (*r*_s_ = −0.693, p = 0.009). A correlation was noticed between α GPS during cold and NRS Unpleasantness at THR (*r_s_* = 0.566, *p* = 0.044) and between δ GPS during pain and PCS (*r_s_* = 0.640, p = 0.018). In NRNA (B, bottom panel), CSI was correlated to HAD_A_ (*r_s_* = 0.740, *p* < 0.001) and to HAD_D_ (*r_s_* = 0.658, *p* < 0.001), the latter being correlated to the other (*r_s_* = 0.599, *p* = 0.003). CSI correlated with ISI (*r_s_* = 0.528, *p* = 0.012). NRS Intensity and NRS Unpleasantness were, respectively, correlated to each other at THR (*r_s_* = 0.732, *p* < 0.001) and at TOL (*r_s_* = 0.723, *p* < 0.001). Pain perception time correlated with HAD_A_ (*r_s_* = −0.534, *p* = 0.022) and pain appearance time correlated with NRS Unpleasantness at THR (*r_s_* = 0.599, *p* = 0.004). In addition, pain perception time correlated with the cold immersion time (*r_s_* = 0.887, *p* < 0.001). A correlation was seen between Hβ GPS at THR and FPQ-9 (*r_s_* = 0.540, *p* = 0.017), and between α GPS during cold (*r_s_* = 0.523, *p* = 0.022) and at THR (*r_s_* = 0.540 p = 0.017) with ISI, between α GPS during pain (*r_s_* = 0.560, *p* = 0.013) and at TOL (*r_s_* = 0.576, *p* = 0.001) with the self-reported sleep duration per night.

It was interesting to comparatively test if and how clinical indicators (especially pain-related behavioral and CPT response indicators) were correlated between them and with EEG markers (especially GABAergic biomarkers) in RA and NRNA (Figure 6). Although there was a large set of significant correlations noticed in the two groups, only some of them persisted after correction for multiple testing (BH method, see the Methods section). Residual significant correlations showed all a large effect size and a *p* < 0.001. We primarily concentrated on them (unless otherwise specified, the effect size and the significance level will be the above-mentioned ones). RA displayed a *negative* correlation between pain appearance time and pain perception time (*r*_s_ = −1.0) and between Lβ GPS at THR and NRS intensity at TOL (*r*_s_ = −0.780, *p* = 0.002). NRNA group showed significant *positive* correlations between CSI and HAD_A_ (*r*_s_ = 0.740), CSI and HAD_D_ (*r*_s_ = 0.658), and between HAD_A_ and HAD_D_ (*r*_s_ = 0.599, *p* = 0.003). In addition, a *positive* correlation existed between cold immersion time and pain perception time (*r*_s_ = 0.887); and between NRS intensity and unpleasantness at THR (*r*_s_ = 0.732) and at TOL (*r*_s_ = 0.723).

In order to allow a broader discussion in such an exploratory study, statistically significant correlations with large effect size, that lost significance after BH correction for multiple testing, were secondarily taken into consideration (Figure 6). In this respect, in the RA group, the PCS was *negatively* correlated with the weekly training duration and HAD_A_, while the latter exhibited a *positive* correlation with ISI. During the CPT, a *negative* correlation was observed between pain appearance time and NRS unpleasantness level and a positive correlation between pain perception time and NRS unpleasantness, both at TOL. A *negative* correlation was found between Lβ GPS at THR and PCS; between Lβ GPS and NRS intensity, both at TOL; as well as between Hβ GPS at THR and both PCS and NRS intensity at TOL. Conversely, a *positive* correlation was observed between α GPS during cold perception and NRS unpleasantness at THR, and between δ GPS during pain perception and PCS. In the NRNA group, a *negative* correlation existed between pain perception time and HAD_A_, while pain appearance time *positively* correlated with NRS unpleasantness at THR. In addition, a *positive* correlation was observed between Hβ GPS at THR and FPQ-9, as well as between α GPS during cold perception and ISI and between α GPS at THR and ISI. Additionally, a *positive* correlation was detected between α GPS during pain perception and at TOL, and the self-reported sleep duration per night.

Overall, the correlational analysis showed a negative set of correlations in RA, in contrast to positive correlations noticed in NRNA. Here, it is interesting to note that significant correlations persisting after correction for multiple testing were coherent with those with large effect size of which significance vanished after BH correction (Figure 6), both in the RA and in the NRNA groups.

## 4 Discussion

### 4.1 General considerations and main findings

This study aimed to explore mechanisms underlying the analgesic effect of PE in pain-free individuals, with a special interest on the GABAergic neurotransmission (potentially involved in EIH and owing to its implication in pain regulation and documented CP-associated modifications). For this purpose, we compared intensively trained endurance athletes with age-adjusted non-trained controls during exposure to a CPT (an appropriate CP experimental procedure). No participant suffered from specific pain disturbance. Additionally, we investigated their pain-related behaviors (central sensitization, fear of pain and catastrophizing features). We made the general hypothesis that the highly trained group would better resist to cold-induced pain and would exhibit an increase in brain GABAergic neurotransmission in addition to more favorable pain-related behavioral profiles.

As stated in the methods, in addition to correlations that remained statistically significant after corrections for multiple testing, only initially significant correlations were mentioned in the result section, and they all displayed a large effect size. Interestingly, most of them were coherent with significant results, suggesting that BH corrections might have been too conservative in this small preliminary study. Therefore, we also discuss them below, but solely in relation with the main results.

The most important finding of this study was the identification of a group of individuals mostly represented in the athlete group (resistant athletes, RA), who, compared to non-resistant non-athletes (NRNA), displayed potentially interesting distinctive characteristics: not only they showed significantly lower fear of pain level, but they better resisted to cold-induced pain, all this in accordance with our working hypotheses. Surprisingly however, RA showed *decreasing* dynamics of GABAergic EEG markers (Hβ and Lβ power), in contrast to the increase noticed not only in other EEG indicators (*α* and *δ* power), but also in all EEG markers in NRNA.

### 4.2 Characterization of RA and differentiation from NRNA

Since RA and NRNA differed mainly by the intensity of their training regime, our results suggest that the observed differences in their pain-related behavior, response to cold-induced pain and GABAergic dynamics could be attributed to long-lasting intensive endurance sport, thereby further supporting our hypothesis. The meaningful element could be the endurance itself or another (unknown) training element not related to endurance (please also refer to the paragraph on the clinical applicability for further discussion). This finding is in accordance with existing literature suggesting reduced pain perception and improved pain-copying strategies upon acute and regular physical training (Ellingson et al., 2012; Naugle et al., 2012; Koltyn et al., 2014; Sluka et al., 2018; Vaegter and Jones, 2020) and confirms that our experimental frame was appropriate to reliably study exercise-induced pain reduction, in particular through characterization of the newly disclosed category of RA.

The RA group, while displaying significantly lower fear of pain than NRNA exhibited non-significant decrease (although moderate in size effect) in other pain-related behavioral indicators (i.e., central sensitization and pain catastrophizing features). The practice of endurance sport is associated with the ambition to go beyond one’s limits, which implies, among other challenges, resisting to potentially painful conditions (Scott and Gijsbers, 1981; Lentini et al., 2021) and even giving them a positive meaning (i.e., not linking them to a potential threat or to a disease) in order to be performant (Geva and Defrin, 2013; Kakiashvili et al., 2016). Lower fear of pain becomes thus fully meaningful in this modified behavioral paradigm. The significantly negative correlation with large effect size between pain catastrophizing features and the number of training hours per week in RA, although disappearing with multiple testing correlation, would further suggest, if confirmed, the link between the pain-related behavioral change and the training regime.

The GABAergic markers (Hβ and Lβ power) were similar at baseline and in all explored perceptions between RA and NRNA. However, not only they differently evolved through key CPT steps compared to other EEG markers (α and δ power) in RA, but their modifications were different between the RA and the NRNA groups. Indeed, a significant interaction was observed between GABAergic markers at cold and pain perceptions, as well as at THR and TOL time-points when comparing the two groups. Careful observation of post-hoc testing results suggests that interaction between cold and pain in Lβ frequency domain could be explained by increase in the NRNA group, and interaction in Hβ domain between THR and TOL by decrease in the RA group. Interestingly, the direction of the remaining GABAergic EEG modifications, although not reaching statistical significance, was similar to significant results (i.e., decrease in RA and increase in NRNA). Given the close similarities noticed in GABAergic decreasing (or non-increasing) trends between cold and pain perception and between THR and TOL, we interpreted them as being part of the same physiological process, and specifically induced by the experiment; highly suggesting differential modifications of the GABAergic signaling in RA and NRNA in response to cold-induced pain.

The GABAergic decrease (or absence of increase) in the presence of cold-induced pain in RA is against our initial working hypothesis that was based on the GAD upregulation subtending EIH and on the decrease of GABAergic signaling and increased brain excitability observed in CP conditions. It appears therefore, at first sight, hard to coherently relate our results to exercise-induced analgesia. However, GABAergic markers were negatively and highly correlated to the sensory pain at TOL (the only pain indicator that was lower in RA than in NRNA in a significant way) and to pain catastrophizing features in RA (although non-significantly upon multiple-testing correction). All these findings highly suggest a GABAergic contribution to reduce experimental pain and to modify pain-copying strategies in RA.

The lowering of GABAergic markers in RA recalls the decrease of many other physiological and metabolic indicators (e.g., heart rate, blood pressure) after several weeks of endurance training (Wilmore et al., 2001), possibly suggesting a similar link between our results and the long-lasting endurance training. In this perspective, the observed decrease could be understood as a counterpart of the neural efficiency reported in highly trained athletes (Li and Smith, 2021). Unfortunately, there is no report of a decreased brain inhibition under regular training conditions in the literature. In contrast, short-term increased brain cortical excitability and decreased GABAergic inhibition have been consistently observed upon acute PE (Lulic et al., 2017; Monda et al., 2017; El-Sayes et al., 2020; Nicolini et al., 2021; Hendy et al., 2022) in association with antinociception and subsequent increase in THR and reduced pain perception in healthy populations (Tang et al., 2009; Pagano et al., 2012; Moloney and Witney, 2014; Granovsky et al., 2019). Interestingly, in the HERITAGE Family study mentioned above (Wilmore et al., 2001), the decrease of metabolic indicators noticed after several weeks of training was enhanced upon additional but more acute and effort-demanding exercise series, suggesting a similarity of effects between the short-term and the long-lasting PE, possibly cumulating one with another. By analogy, this could also apply to brain GABAergic signaling modifications in RA. We could not verify this assumption because RA were not submitted to acute exercise in our study. In case our hypotheses were confirmed, the GABAergic decrease could mediate regular exercise-induced analgesia (Ellingson et al., 2012; Sluka et al., 2018; Vaegter and Jones, 2020) as well. In this scenario, exercise-induced lowering of the GABAergic signaling could be considered as an adaptive beneficial mechanism against pain and pain-related aberrant behaviors, possibly related to the above-mentioned neural efficiency.

Nevertheless, it is difficult to reconcile this interpretation with the antinociception associated with GAD upregulation in experimental animal models upon acute PE (Kami et al., 2016; Senba and Kami, 2020). Accordingly, there are indications that endurance athletes increase their resting-state EEG β power in all brain areas when submitted to acute PE at maximal load, irrespective of their neural efficiency (Ludyga et al., 2016). In this perspective, the observed lowering of GABAergic markers in an acute exercise context would possibly be part of training-related body adaptations, allowing a broader range of performance increase under (acute) extreme training conditions (Bjørnstad et al., 1993).

On the other hand, the increased GAD activity and expression (and the subsequent enhancement of the GABAergic signaling) were observed in neuropathic pain animal models and were interpreted as a therapeutic strategy rescuing deficient GABAergic neurotransmission. Furthermore, in patients suffering from fibromyalgia-related CP, a decrease in Hβ functional connectivity was correlated to the affective pain in the basolateral area of the amygdala (interpreted as participating to CP pathological mechanisms), whereas Lβ increased as a function of pain intensity in the prefrontal cortex (seen as a compensatory mechanism) (Makowka et al., 2023).

Overall, these results and related discussions suggest that exercise-related modifications in brain GABAergic signaling could be different not only between differently trained healthy populations, but possibly also between pain-free and pain-affected individuals. Consequently, the GABAergic decrease, while being beneficial upon long-lasting endurance training (regardless of its interpretation), could be part of pathophysiological mechanisms in CP conditions.

Considering these multifaceted GABAergic changes in pain-free and CP conditions, and the supposedly antinociceptive effect of PE, it was interesting to further discuss differences between RA and NRNA regarding their respective associations between clinical variables and between clinical and EEG indicators. From a clinical standpoint, pain-catastrophizing features negatively correlated with the anxiety score and the latter to the sleep dysfunction score in RA, suggesting that the known association between both anxiety (and depression), and pain catastrophizing in CP patients (Dong et al., 2020) could be disrupted in RA. A positive correlation between central sensitization and mood indicators was present in NRNA, similarly to reports in CP syndromes (Proença et al., 2021; Valera-Calero et al., 2022; Fernández-de-Las-Peñas et al., 2023). The NRNA appeared thus to have a pain-related behavior closer to CP patients and differed from the improved behaviors noticed in RA.

During the CPT, pain sensitivity was negatively correlated to pain resistance (indicated by the pain perception time) in RA, implying that the more the latter were sensitive to the induced pain, the more they resisted to it, as if higher sensitivity to induced pain primed or prepared to better resist to it. Supporting this interpretation, pain intensity at TOL was indeed significantly lower in RA. This behavioral mechanism of pain resistance seemed to engage the affective dimension of pain (which increased along with pain resistance but was reversely associated with pain sensitivity), consistently with the involvement of affective inputs and related brain pathways reported in EIH (Kami et al., 2022). In NRNA, however, the cold immersion time positively correlated to the pain perception time, which tended to minimize the role of the pain appearance time. Sensory and affective pain indicators evolved in a linear way between THR and TOL in NRNA (but not in RA), with positive correlations noticed between them at both experimental steps. Of notice, pain resistance was negatively associated to the anxiety score in NRNA, but not in RA. These observations suggest that, compared to NRNA, RA decoupled their THR from their TOL pain level in order to better resist to high pain level. In fact, TOL experimental step exhibited the most meaningful differences between RA and NRNA (sensory pain level) and affective pain level associations (negative with pain sensitivity and positive with pain resistance) in RA.

As stated above, NRNA showed an increase in one GABAergic EEG marker from cold to pain perception with significant interaction, contrary to RA. Furthermore, NRNA displayed positive correlation between one GABAergic marker and fear of pain, whereas correlations between GABAergic markers and pain catastrophizing, as well as with pain intensity at TOL were systematically negative in RA.

These observations suggest that in NRNA, GABAergic increase paralleled perceived pain and pain-related fear; as if NRNA simply adapted their GABAergic signaling and behavior to pain, while RA went a step beyond, decreasing their GABAergic neurotransmission as part of mechanisms attenuating their maximal sensory pain level during the CPT and counteracting meaningful pain-related behavior (namely pain catastrophizing). In this new interpretative frame, non-trained CP patients would be unable to increase their GABAergic signaling as a function of experienced pain, due to chronic-pain-related pathophysiological changes. This plausible dual role of brain hyperexcitability (and therefore of GABAergic decrease) in trained pain-free population versus in CP patients further illustrates the above-hypothesized multifaceted role of GABAergic changes according to the training regime and to the existence of pain. Interestingly, the increase in brain-derived neurotrophic factor (BDNF), which is considered as a marker of a “virtuous” brain neuroplasticity (e.g., following PE; Nicolini et al., 2021), participates at the same time to the (neuropathic) pain pathological neuroinflammatory cascade resulting in the nervous hyperexcitability (Sikandar et al., 2018; Thakkar and Acevedo, 2023). Thus, nociception and pain regulation players could differently change according to homeostatic conditions and their modifications should be interpreted with caution.

### 4.3 Potential clinical applicability and translation of obtained results

The present study was conducted in highly trained athletes (≥ 7 h of weekly training), who do not represent the typical profile of CP patients (subject to aberrant behavior toward pain and movement; see the introduction; Vlaeyen and Linton, 2000; Crombez et al., 2012; Zale and Ditre, 2015), or even the trends of PE intensity in the general population. Therefore, one important question is to know whether obtained results would be applicable in populations targeted by PE as an analgesic therapy.

At this point, there is no direct indication that our results would apply to non-athlete populations (including CP patients). However, it should be remembered that resistant individuals were also present among non-athletes, albeit at a lower proportion. We could not characterize them for this reason, but in case they would display a similar profile as RA, this would open a possibility to translate the observations made in RA to less trained individuals. On the other hand, if we take into consideration the observed involvement of the GABAergic neurotransmission associated with modified pain response and pain-related behaviors, recent data show that acute highly- versus moderately intense exercise do not seem to differ regarding brain excitability in low fit individuals (El-Sayes et al., 2020). Further, when submitted to acute PE sessions upon several week-physical training, low fit individuals exhibit brain excitability decrease as measured by an indicator of brain cortical inhibition (Lulic et al., 2017; El-Sayes et al., 2020). These observations suggest the combination of acute PE and prior long-lasting training as the most suitable regime to impact brain excitability (thereof, brain GABAergic signaling) in non-athletes, provided that obtained modifications are associated with the desired analgesia (which was not investigated in above-mentioned studies). In summary, the response to acute training (which was not measured in this study) and the issue of a minimal necessary dose or a dose-dependent effect of PE eliciting the beneficial modification of pain response should be further investigated prior to translation into clinical practice. Additionally, the acute pain, which corresponds better to the physiological nociceptive model (Sneddon, 2018) and would be more suitable to the experimental pain model in general, should also deserve some interest in the perspective of EIH, and more broadly to the analgesic effect of PE.

Another interesting question is how meaningful the endurance component of the training to RA resistance to cold-induced pain is (and hence possibly to the effectiveness of PE-induced analgesia). In comparison to strength athletes, endurance athletes display significantly higher tolerance to pain and lower fear of pain (Assa et al., 2019), which supports the importance of endurance, although one cannot exclude the possibility that another independent training factor (to be further investigated) may play a role. In complement, the implication of the GABAergic signaling would open a way for synergy between endurance training and GABA-modifying analgesic treatments.

The variability of EIH observed among chronic pain patients (Rice et al., 2019) should also be analyzed under the lenses of possible differences in brain GABAergic dysfunction between CP syndromes, calling for a mechanism-based classification of CP diseases before applying a given therapy modifying a precise pathway (here, the GABAergic signaling). Thus, future studies investigating exercise-induced GABA-mediated analgesia in different CP syndromes should also compare the involvement of the GABAergic neurotransmission in the pathological process to better target responsive individuals (or syndromes).

## 5 Study limitations

Despite interesting observations discussed above, and for the potential clinical applications of our findings, a number of limitations have to be stated. First, because our data were purely experimental and collected in pain-free individuals, their confirmation in pain-diseased patients should be warranted before effective clinical translation. Second, despite a moderate to high theoretical study power, we cannot exclude the role of the modest sample size in the variability of our results, especially when data lost significance upon correction from multiple testing or showed no significant differences or correlations. Third, excluding females from the sample, although reducing the variability of collected data by avoiding sex-related bias in pain response (Bartley and Fillingim, 2013), limited generalization and translation of obtained results, considering also that women are more affected by any type of pain than men (Osborne and Davis, 2022). Thus, our findings should be confirmed in more inclusive and larger samples. In particular regarding the sample size estimation, the key endpoints should be significant differences between resistant athletes and non-resistant non-athletes, regarding clinical (mainly pain unpleasantness and, albeit to a lesser extent, pain intensity) and EEG data (Lβ and Hβ GPS) modifications in response to the CPT. More specifically, their differences at THR and TOL, as well as differences in their respective modifications between THR and TOL. Most probably, a compromise should be found between the clinical and the electrophysiological perspective, given the difference of sample size derived from similar statistical power in existing studies (see the discussion above). Fourth, athletes were overall significantly younger than non-athletes. Although this difference was not anymore significant when comparing RA to NRNA, it could account for some differences observed between athletes and non-athletes (and more specifically between RA and NRNA). Thus, obtained results should be confirmed comparing equally aged groups in order to exclude a potential age-related bias.

In addition, we made a number of indirect assumptions based on studies of brain hyperexcitability performed using a different method (transcranial magnetic stimulation) (Mooney et al., 2016; Monda et al., 2017; Moscatelli et al., 2021) or based on the assumed analogy between metabolic changes upon long-lasting endurance training and our results (Wilmore et al., 2001), while we did not directly assess them, neither have we found such indications in the literature. We should therefore be cautious about stated similarities. Finally, our experimental settings could be subject to a number of biases. Immersing the participant’s hand in warm water before proceeding to the CPT could constitute a conditioning step vanishing some discriminating features between or within the studied groups during the experiments. Also, we evaluated behavioral data only at baseline, not during or just upon the CPT, while they could be measurably modified and further influence pain response. Data from RA were most probably biased by the imposed limit of 4 min immersion time, which could impact all performed analyses (Årnes et al., 2023).

## 6 Conclusion

The whole idea behind this study was to better understand mechanisms by which PE would induce analgesia by comparatively investigating pain-related behavior and response to experimental cold-induced pain of highly trained athletes and non-trained individuals. Our results suggest that the most resistant athletes improve their pain-related behavioral features and seem to dissociate the latter from mood and sleep dysfunction. Furthermore, resistant athletes appear more resistant to experimental cold-induced pain, with associated reduction in GABAergic neurotransmission.

Despite its limitations, this study constitutes one of the first investigations enlightening mechanisms underlying exercise-induced hypoalgesia. Furthermore, although the decreased brain GABAergic neurotransmission goes against our working hypothesis, we propose a coherent interpretation by comparatively discussing differences in clinical and GABAergic indicators between the two studied groups, and in light of the current knowledge about the multiple effects of physical exercise (e.g., metabolic changes, modifications in brain excitability). Subsequently, a multimodal profile of GABAergic changes according to homeostatic conditions (namely, the training regime and possibly the presence or absence of pain) is hypothesized. Out of it, a preliminary orientation on the therapeutic applicability of exercise-induced analgesia, based on the GABAergic neurotransmission, can be further investigated in future studies.

## Data availability statement

The raw data supporting the conclusions of this article will be made available by the authors, without undue reservation.

## Ethics statement

The studies involving humans were approved by Ethical Committee of Vaud, Switzerland. The studies were conducted in accordance with the local legislation and institutional requirements. The participants provided their written informed consent to participate in this study.

## Author contributions

FP: Conceptualization, Data curation, Formal analysis, Investigation, Methodology, Project administration, Writing – original draft, Writing – review & editing. MM: Data curation, Formal analysis, Methodology, Software, Validation, Writing – review & editing. MDP: Data curation, Software, Validation, Writing – review & editing, Methodology. JC: Conceptualization, Data curation, Formal analysis, Funding acquisition, Investigation, Methodology, Project administration, Supervision, Validation, Writing – original draft, Writing – review & editing.

## Figures and Tables

**Table:** 

α	Alpha
ANOVA	Analysis of Variance
BDNF	Brain-Derived Neurotrophic Factor
BH	Benjamin Hochberg
BMI	Body Mass Index
CP	Chronic Pain
CPT	Cold Pressure Test
CSI	Central Sensitization Index
δ	Delta
EEG	Electroencephalography
EIH	Exercice-Induced Hypoalgesia
FPQ-9	Fear of Pain Questionnaire (9 Items)
GAD	Glutamate Acid Decarboxylase
GABA	Gamma-Aminobutyric Acid
GPS	Global Power Spectrum
HAD	Hospital Anxiety and Depression
HAD_A_	Hospital Anxiety and Depression subscore for Anxiety
HAD_D_	Hospital Anxiety and Depression subscore for Depression
Hβ	High Beta
Hz	Herz
IQR	Interquartile Range
ISI	Insomnia Severity Index
Lβ	Low Beta
NRA	Non-Resistant Athletes
NRNA	Non-Resistant Non-Athletes
NRS	Numeric Rating Scale
NRS_I_	Numeric Rating Scale for pain Intensity
NRS_U_	Numeric Rating Scale for pain Unpleasantness
PAT	Pain Appearance Time
PCS	Pain Catastrophizing Scale
PE	Physical Exercise
PPT	Pain Perception Time
THR	Threshold
TOL	Tolerance
RA	Resistant Athletes
SD	Standard Deviation
μV	Micro Volt
VO_2max_	Maximal Oxygen consumption

## Footnotes

^1^https://www.nitrc.org/projects/cleanline
