# Supplementary material for: Response to experimental cold-induced pain discloses a resistant category among endurance athletes, with a distinct profile of pain-related behavior and GABAergic EEG markers: a case–control preliminary study
Source: Front Neurosci. 2024 Jan 15;17:1287233. doi: 10.3389/fnins.2023.1287233 (PMC10822956; doi:10.3389/fnins.2023.1287233)
Supplement: Supplementary file 1 [file Data_Sheet_1.docx]

**Supplementary table 1. EEG power modifications according to perceptions and response to the CPT**

| **GPS [μV^2^/Hz]** | **Perceptions** | **Hß** | **Lß** | **α** | **δ** |
| --- | --- | --- | --- | --- | --- |
| **RA**  **(n=13)** | **Baseline** | 111 (50.8)  (46.8 – 190) | 211 (53.2)  (101 – 419) | 1636 (1545)  (257 – 3978) | 1157 (584)  (417 – 1857) |
|  | **Warm** | 189 (285)  (91.5 – 568) | 301 (182)  (142 – 472) | 422 (227)  (154 – 1322) | 1677 (921)  (840 – 3672) |
|  | **Cold** | 275 (207)  (72.8 – 782) | 288 (159)  (111 – 575) | 389 (371)  (164 – 1311) | 1443 (915)  (687-3491) |
|  | **Pain** | 170 (180)  (125 – 642) | 270 (98.1)  (125 – 642) | 477 (774)  (195 – 2262) | 1264 (1109)  (618 – 2919) |
| **NRA (n=13)** | **Baseline** | 107 (95.7)  (48.6 – 287) | 194 (103)  (106 – 968) | 1575 (2169)  (154 – 3701) | 935 (227)  (673 – 2070) |
|  | **Warm** | 295 (155)  65.8 – 489) | 311 (148)  (106 - 641) | 496 (324)  (259 – 860) | 1580 (599)  (1062 – 2909) |
|  | **Cold** | 403 (272)  (111 – 606) | 302 (159)  (160 – 654) | 366 (204)  (185 – 668) | 1142 (834)  (661 - 3569) |
|  | **Pain** | 376 (166)  (133 – 825) | 364 (254)  (179 – 652) | 427 (347)  (228 – 1180) | 1413 (761)  (808 – 6235) |
|  | **THR** | 524 (632)  (77.7 – 994) | 485 (312)  (106 – 697) | 451 (427)  (164 – 971) | 1285 (1016)  (377 – 15841) |
|  | **TOL** | 314 (203)  (173 – 960) | 354 (99.5)  (225 – 665) | 466 (256)  (192 – 1138) | 1372 (817)  (779 – 6458) |
| **NRNA (n=19)** | **Baseline** | 117 (76.8)  (41.2 – 235) | 258 (296)  (74.3 – 908) | 805 (1875)  (89.6 – 12460) | 813 (721)  (346 – 4110) |
|  | **Warm** | 228 (127)  (129 – 874) | 239 (129)  (131 – 593) | 359 (224)  (197 – 1440) | 1275 (558)  (497 - 3079) |
|  | **Cold** | 244 (226)  (105 – 915) | 296 (184)  (137 – 629) | 355 (274)  (154 – 1026) | 1120 (722)  (282 – 4092) |
|  | **Pain** | 310 (264)  (130 – 603) | 345 (199)  (130 – 603) | 494 (354)  (226 – 2154) | 1452 (1091)  (518 - 5347) |

Data are given as median (InterQuartile Range, IQR) and range (min-max). Electroencephalographic (EEG) powers are expressed as Global Power Spectra (GPS, μV^2^/Hz = microvolt^2^/Herz) in different frequency ranges (Hβ=High beta (20-30 Hz), Lβ=Low beta (13-20 Hz), α=alpha (8-12 Hz), δ=delta (2-4 Hz)); at baseline, and during warm, cold and pain perceptions. According to their resistance to pain, participants were categorized as Resistant Athletes (RA), Non-Resistant Athletes (NRA) and Non-Resistant Non-Athletes (NRNA) (see the result section in the main manuscript for more details). In addition, the GPS at threshold (THR) and tolerance (TOL) are showed for NRA.
